# Modulation of foraging-like behaviors by cholesterol-FGF19 axis

**DOI:** 10.1186/s13578-023-00955-2

**Published:** 2023-02-02

**Authors:** Alyssa Huang, Matthew T. Maier, Eirini Vagena, Allison W. Xu

**Affiliations:** 1 Diabetes Center, University of California, San Francisco, CA 94143 USA; 2 Department of Anatomy, University of California, San Francisco, CA 94143 USA

**Keywords:** Foraging, Dietary cholesterol, AgRP, FGF19

## Abstract

**Background:**

Foraging for food precedes food consumption and is an important component of the overall metabolic programming that regulates feeding. Foraging is governed by central nervous system neuronal circuits but how it is influenced by diet and hormonal signals is still not well understood.

**Results:**

In this study, we show that dietary cholesterol exerted suppressive effects on locomotor activity and that these effects were partially mediated by the neuropeptide Agouti-related protein (AgRP). High dietary cholesterol stimulated intestinal expression of fibroblast growth factor 15 (*Fgf15*), an ortholog of the human fibroblast growth factor 19 (*FGF19*). Intracerebroventricular infusion of FGF19 peptide reduced exploratory activity in the open field test paradigm. On the other hand, the lack of dietary cholesterol enhanced exploratory activity in the open field test, but this effect was abolished by central administration of FGF19.

**Conclusions:**

Experiments in this study show that dietary cholesterol suppresses locomotor activity and foraging-like behaviors, and this regulation is in part mediated by AgRP neurons. Dietary cholesterol or the central action of FGF19 suppresses exploratory behaviors, and the anxiogenic effects of dietary cholesterol may be mediated by the effect of FGF19 in the mouse brain. This study suggests that dietary cholesterol and intestinal hormone FGF15/19 signal a satiating state to the brain, thereby suppressing foraging-like behaviors.

**Supplementary Information:**

The online version contains supplementary material available at 10.1186/s13578-023-00955-2.

## Introduction

In nature, food availability is highly unpredictable and often limited. As such, free-living animals spend considerable time and effort foraging for food. These traits are possessed by animals across the animal kingdom, including our human ancestors. Even in the modern era, excessive food seeking behaviors are displayed by people with Prader-Willi Syndrome or MC4R-deficiency [1–5]. More generally, individuals who experience food insecurity also exhibit enhanced food seeking behaviors, and they are prone to the development of obesity [6–9]. Thus, foraging behaviors are essential for survival and these traits are highly conserved in animal species including humans.

Food-seeking precedes food consumption, and these two processes are often tightly coupled. Neurons expressing agouti-related protein (AgRP) are located in the mediobasal hypothalamus and they co-express neuropeptide Neuropeptide Y (NPY). Food deprivation potently stimulates both AgRP expression and neuronal activity. AgRP neurons are widely recognized for their ability to promote food intake, as optogenetic or chemogenetic stimulation of AgRP neurons leads to voracious feeding, and acute ablation of these neurons leads to severe anorexia [10–12]. Paradoxically, release of NPY, but not AgRP, from AgRP neurons mediates the orexigenic effects induced by acute activation of these neurons [13, 14]. Notably, activation of AgRP neurons also stimulates locomotor activity and foraging behaviors beyond food intake [11, 15–17], and it exerts anxiolytic effects [18]. These findings suggest that foraging-like behaviors are fundamental innate response to food availability, and that AgRP neurons, key regulators of feeding, are involved in this process.

To date, the environmental factors that influence foraging-like behaviors are still not well understood. We recently show that altering dietary cholesterol intake affects hypothalamic AgRP expression and dietary preference [19]. Cholesterol is essential for life, but energetically costly to synthesize and cytotoxic when in excess. Thus, cholesterol production is under extreme tight regulation. The body normally obtains cholesterol from two sources: the diet and de novo biosynthesis mainly by the liver. When dietary cholesterol is high (i.e.  ≥ 1%), hepatic cholesterol synthesis is suppressed by robust negative feedback mechanisms [20]. However, when dietary cholesterol is absent, hepatic cholesterol synthesis is markedly stimulated to meet the body’s need [21]. Cholesterol is the precursor for bile acids, which are mainly synthesized in the liver and stored in the gallbladder. Bile acids are released postprandially into the small intestine where they emulsify lipids for absorption by enterocytes. In the distal small intestine, bile acids activate nuclear hormone receptor farnesoid X receptor (FXR) in enterocytes to stimulate the synthesis and release of FGF15/19 into circulation (FGF15 is the mouse ortholog of human FGF19). FGF15/19 in turn binds to FGFR4 in hepatocytes to suppress the synthesis of bile acids [22, 23]. Notably, multiple lines of evidence demonstrate that FGF15/19 also acts in the brain to affect neuronal functions [24–28]. In this study, we demonstrate that high dietary cholesterol and FGF19 suppress foraging-like behaviors, and that the effects of dietary cholesterol on locomotor activity are partially mediated by AgRP.

## Results

### Locomotor activity increases with food deprivation, and it depends on AgRP in a diet-specific manner

Foraging-like behaviors increase with food deprivation, a condition that is associated with enhanced expression of hypothalamic AgRP [29]. We thus evaluated if locomotor activity was affected by the loss of AgRP in either *ad lib* fed or fasting condition. To this end, weight-matched *Agrp*^+*/*+^ and *Agrp*^*–/–*^ mice were individually housed in chambers of the Comprehensive Lab Animal Monitoring System (CLAMS). Locomotor activity and other metabolic parameters were measured. It has been previously established that a 24 h acclimation period is sufficient to habituate for measurement of locomotor activity [30], so data generated on the first day in the CLAMS were excluded from the analysis.

**Fig. 1:**
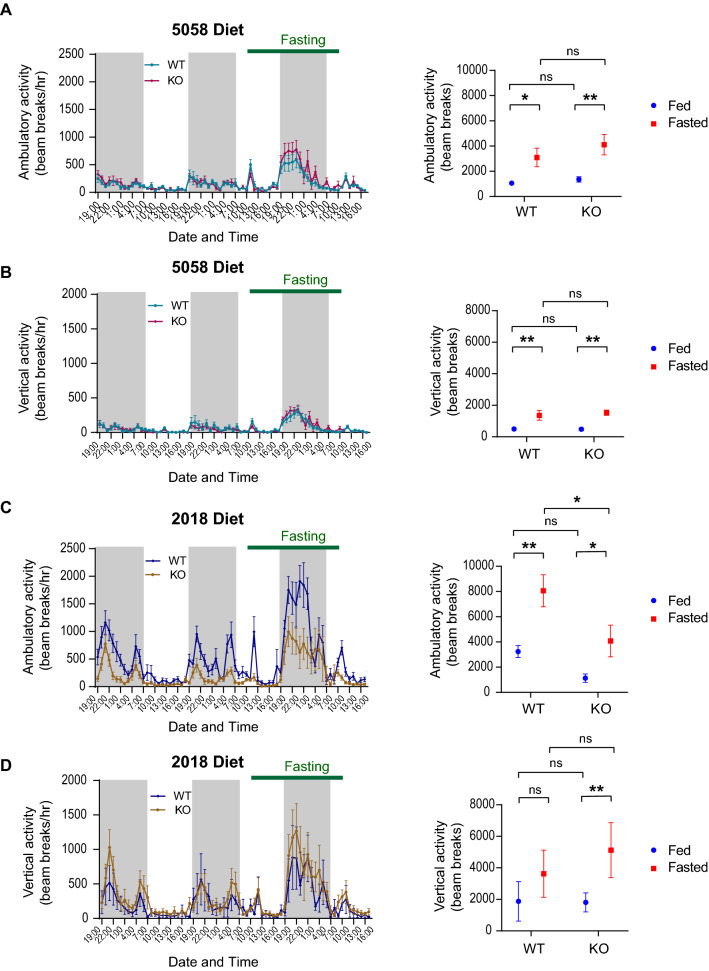
Locomotor activities increase with food deprivation and are dependent on AgRP in a diet-specific manner. **A–B** Adult male *Agrp*^+*/*+^ and *Agrp*^*–/–*^ mice (n = 6 per group; age 3 months) were placed in CLAMS at room temperature and *ad lib* fed with chow with cholesterol (Lab Diet 5058), and subsequently fasted for 24 h, as indicated by green line. In CLAMS while *ad lib* fed with cholesterol-deficient diets (Teklad 2018) at room temperature. Sum of ambulatory **A** or vertical activities **B** during the most active phase of the dark cycle (7PM to 1AM) were compared. **C-D** A separate group of adult male *Agrp*^+*/*+^ and *Agrp*^*–/–*^ mice (n = 7–9 per group; age 5–6.5 months) were placed in CLAMS while *ad lib* fed with cholesterol-deficient diets (Teklad 2018) at room temperature. Sum of ambulatory **C** or vertical activities **D** during the most active phase of the dark cycle (7PM to 1AM) were compared. Data from second *ad lib* fed day in the CLAMS were compared with those from the equivalent time during the fasting period. Gray-shaded areas indicate dark cycles. *Ns* not significant. *p < 0.05. **p < 0.01. ***p < 0.001 by 2-WAY ANOVA with repeated measures

When mice were fed with a cholesterol-containing chow diet (Lab Diet 5058; cholesterol 200 ppm), a 24 h fast significantly stimulated both ambulatory (walking) and vertical activity (rearing and standing), the elevation only manifested in the dark cycle, but not in the light cycle within the fasting period (Fig. 1A–B). *Agrp*^+*/*+^ and *Agrp*^*–/–*^ mice behaved similarly (Fig. 1A–B). Paradoxically, when mice were fed with a cholesterol-free chow diet (Teklad 2018, Envigo), higher levels of ambulatory (Fig. 1A, C) and rearing locomotor activity (Fig. 1B, D) were observed. Notably, compared to *Agrp*^+*/*+^ mice, *Agrp*^*–/–*^ mice exhibited significantly lower levels of ambulatory activity during the fasting period (Fig. 1C). Taken together, these results reinforce the notion that food deprivation, coupled with a nocturnal environment, promotes locomotor activity that resembles foraging-like behaviors. These results also suggest that AgRP is required for the control of ambulatory activity, but this function is manifested only under specific dietary conditions.

### Locomotor activity is suppressed by dietary cholesterol, and this effect is partly governed by AgRP

Given the above results, we investigated whether dietary cholesterol may affect locomotor activity, and if there is a functional interaction between dietary cholesterol and AgRP function. To minimize animal-to-animal variability and differences in various diets, we examined locomotor activity in the same mice that were fed with identical diets differing only in cholesterol contents.

**Fig. 2:**
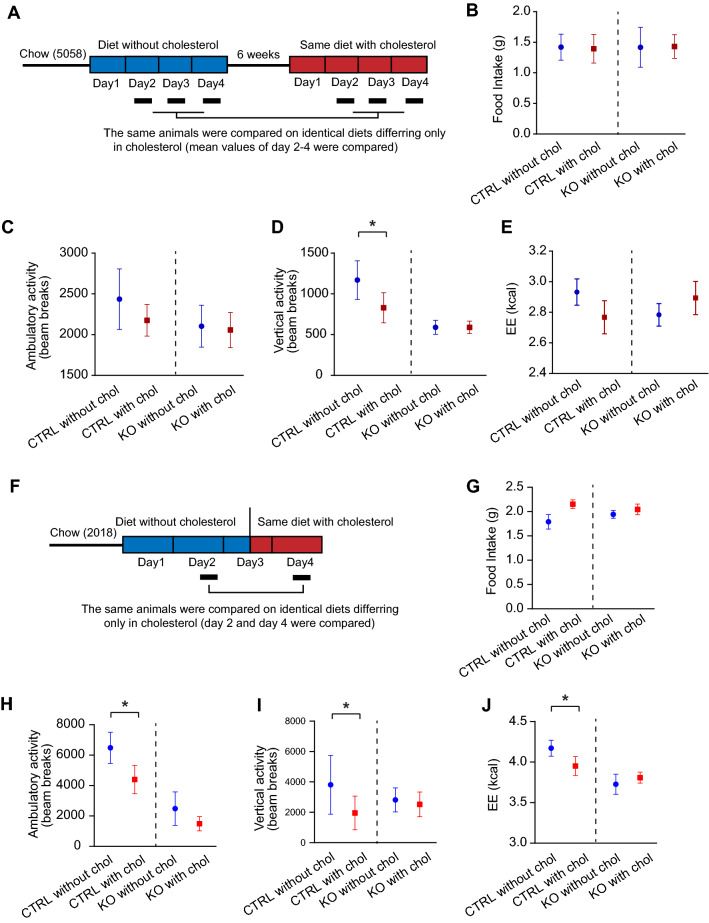
Locomotor activities are suppressed by dietary cholesterol, and this effect is partly governed by AgRP. **A** Experimental procedure. Briefly, a cohort of 7-month-old male *Agrp*^+*/*–^ (n = 6) and *Agrp*^*–/–*^ (n = 6) mice were transitioned from chow (Lab Diet 5058; cholesterol 200 ppm) to a cholesterol-free HFD diet (60% Kcal fat, 30.4% Kcal Carb, 9.6 kcal% protein, < 10 ppm cholesterol) and underwent metabolic measurements in CLAMS under thermoneutrality. The procedure was repeated 6 weeks later only this time the same mice were fed with the same diet containing cholesterol (280 ppm). **B** Food intake, **C** ambulatory activity, **D** vertical activity and **E** energy expenditure during the early night cycle (7PM-1AM) were compared between the two diets. **F** In a separate experiment, adult male *Agrp*^+*/*+^ and *Agrp*^*–/–*^ mice (n = 7–9 per group; age 4–6 months old), maintained on a cholesterol-free chow diet (Teklad 2018), were placed in CLAMS at room temperature and *ad lib* fed with the same diet for first 2 days and then switched to the same diet supplemented with 1% cholesterol. **G** Food intake, **H** ambulatory activity, **I** vertical activity and **J** energy expenditure during the early night cycle (7PM-1AM) were compared. *p ≤ 0.05. **p ≤ 0.01. ***p ≤ 0.001 by two-way ANOVA with repeated measures

*Agrp*^*–/–*^ and control littermates were first given a custom cholesterol-free diet and placed in the CLAMS for the measurement of food intake and locomotor activity. After a 6 week resting period, the same mice were given the same diet as in the first CLAMS run but this time supplemented with 280 ppm cholesterol. Food intake and locomotor activity were measured by CLAMS (Fig. 2A). The addition of dietary cholesterol did not affect food intake (Fig. 2B). There was a trend towards decrease in ambulatory activity in the presence of dietary cholesterol but did not reach statistical significance (Fig. 2C). However, addition of dietary cholesterol led to significant decrease of the rearing activity in the control but not in the *Agrp*^*–/–*^ mice (Fig. 2D). Energy expenditure was not significantly different (Fig. 2E).

In a separate experiment, a different cohort of weight-matched *Agrp*^*–/–*^ and control mice were placed in the CLAMS and fed with a cholesterol-free diet (Teklad 2018, Envigo). After locomotor activity was recorded, mice were given the same diet but supplemented with 1% cholesterol, which mimics cholesterol content in an egg yolk (Fig. 2F). Food intake was not significantly affected by dietary cholesterol (Fig. 2G). In contrast, both ambulatory and rearing activity were reduced after consuming high dietary cholesterol in wild type mice but not the mutant mice (Fig. 2H-I). Similar to locomotor activity, energy expenditure was also reduced in control mice that consumed high dietary cholesterol and not in mutant mice (Fig. 2J). Together, these results suggest that high dietary cholesterol attenuates locomotor activity and that these effects are partially mediated by neuropeptide AgRP.

The differences in ambulatory activities of control mice in Fig. 2C, D and H, I are most likely due to the differences in diets the mice were chronically maintained prior to the start of the CLAMS experiment. Mice for Fig. 2A–E were maintained on cholesterol-containing diet (Lab Diet 5058) before they were switched to a cholesterol-deficient diet for CLAMS study. In contrast, mice in Fig. 2F–J were maintained with a cholesterol-free diet (Teklad 2018) and were continued with the same cholesterol-free diet in the CLAMS. As shown in Fig. 1, mice that were fed with cholesterol-containing diet (Lab Diet 5058) show marked reduction of ambulatory activities compared with mice that were fed with cholesterol-free diet (Teklad 2018). Thus, the differences in ambulatory activities of the control mice in Fig. 2C, D and H, I are consistent with results shown in Fig. 1, suggesting that chronic consumption of cholesterol-free diets enhances basal ambulatory activities.

### Dietary cholesterol results in increased FGF15 expression in the distal small intestine

**Fig. 3:**
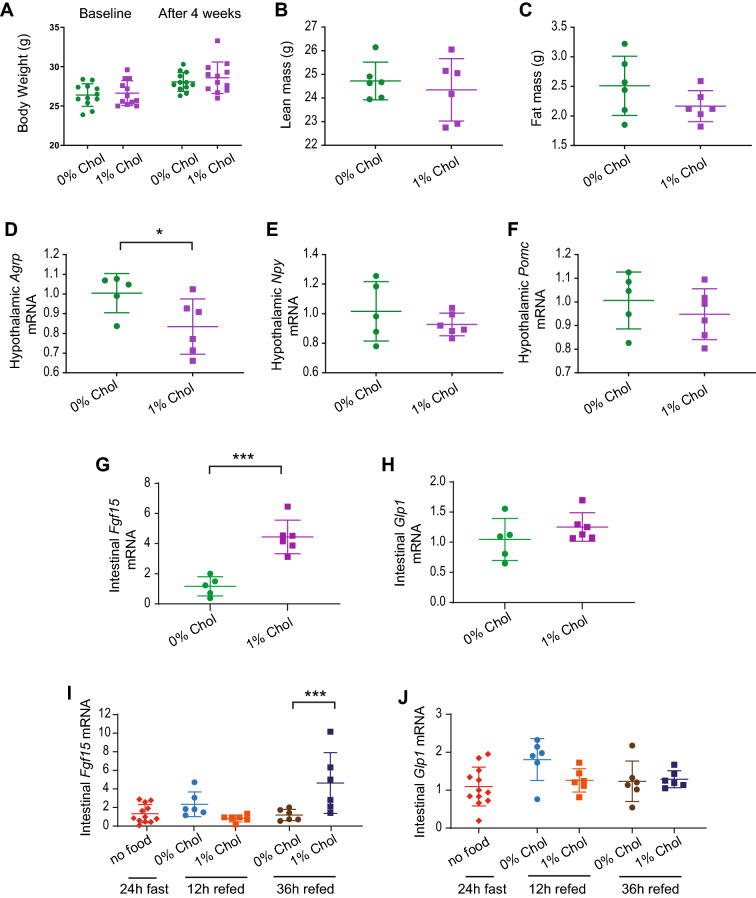
Dietary cholesterol results in increased FGF15 expression in the distal small intestine. **A–C** 10-week-old male C57BL/6 J mice were weight-matched (n = 8 per group) and divided into 2 groups with one group fed with cholesterol-deficient diet (Teklad 2018) and the other with the same diet supplemented with 1% cholesterol for 4 weeks. There were no differences in body weight, lean or fat mass. **D–H** Brain and small intestines were harvested at the same time under *ad lib* fed conditions. Semi-quantitative real-time RT-PCR was carried out on the hypothalamus and distal small intestine. **I–J** 10-week-old male C57BL/6 J mice were divided into several weight-matched groups, fed with cholesterol-deficient diets (Teklad 2018) for 48 h, fasted for 24 h (n = 12) and sacrificed, or fasted and refed with the same diet with or without cholesterol supplement (1%) for 12 h (n = 6 per diet) or 36 h (n = 6 per diet). Distal small intestinal tissue was harvested and mRNA expression for *Fgf15* and *Glp1* mRNA was examined. *p ≤ 0.05. **p ≤ 0.01. ***p ≤ 0.001 by 2-WAY ANOVA

It has been previously shown that infusion of FGF19 in the brain suppresses *Agrp* and *Npy* mRNA expression and inhibits AgRP neuronal activity [27]. Since cholesterol is the precursor of bile acids, known to stimulate *Fgf15/FGF19* intestinal expression, we examined whether cholesterol affected *Fgf15* expression in the mouse distal small intestine. To this end, mice that were fed with diets differing only in the cholesterol content (0% versus 1%) were analyzed for various gene expression. Body weight, lean or fat mass were not affected upon the consumption of the diets after 4 weeks (Fig. 3A–C). However, high dietary cholesterol moderately suppressed expression of *Agrp*, but not *Npy* or *Pomc*, in the hypothalamus (Fig. 3D–F). Notably, high dietary cholesterol caused marked elevation of intestinal *Fgf15* mRNA expression, but did not alter *Glp1* expression (Fig. 3G–H). Intestinal *Fgf15* mRNA expression was also stimulated 36 h after refeeding in mice that consumed the diet supplemented with 1% cholesterol, but not in the mice that were refed the same diet but without cholesterol (Fig. 3I). Intestinal *Glp1* expression during refeeding was not affected by dietary cholesterol content (Fig. 3J). Taken together, these results suggest that dietary cholesterol has potent stimulatory effects on intestinal FGF15/19 expression.

### Dietary cholesterol and FGF19 action in the brain suppresses exploratory behavior

Foraging for food requires coordinated actions of locomotion and willingness to explore in an unfamiliar environment. Open field test is a widely used research tool to evaluate exploratory and anxiety-like behaviors in small animals [31, 32]. Mice have a natural tendency to avoid the center of the open field, which poses a high risk of being exposed to predators in nature. Thus, the number of entries and the time spent in the center of the open field reflect the levels of anxiety and exploratory activity. Of note, by using the open field test, activation of AgRP neurons has been shown to exert anxiolytic effects [18]. Since AgRP neurons release neuropeptide Y and GABA in addition to AgRP, we evaluated whether AgRP plays an indispensable role in mediating the anxiolytic effects of AgRP neurons. To this end, a cohort of *Agrp*^+*/*+^ and *Agrp*^*–/–*^ mice, maintained with the cholesterol-free chow (Teklad 2018), were subjected to open field test. No differences were observed between control and mutant mice in any of the parameters examined (Additional file 1: Figure S1). This result suggests that AgRP is not required for the anxiolytic effects of AgRP neurons or that this function of AgRP is readily compensated by other components of the AgRP neurons.

**Fig. 4:**
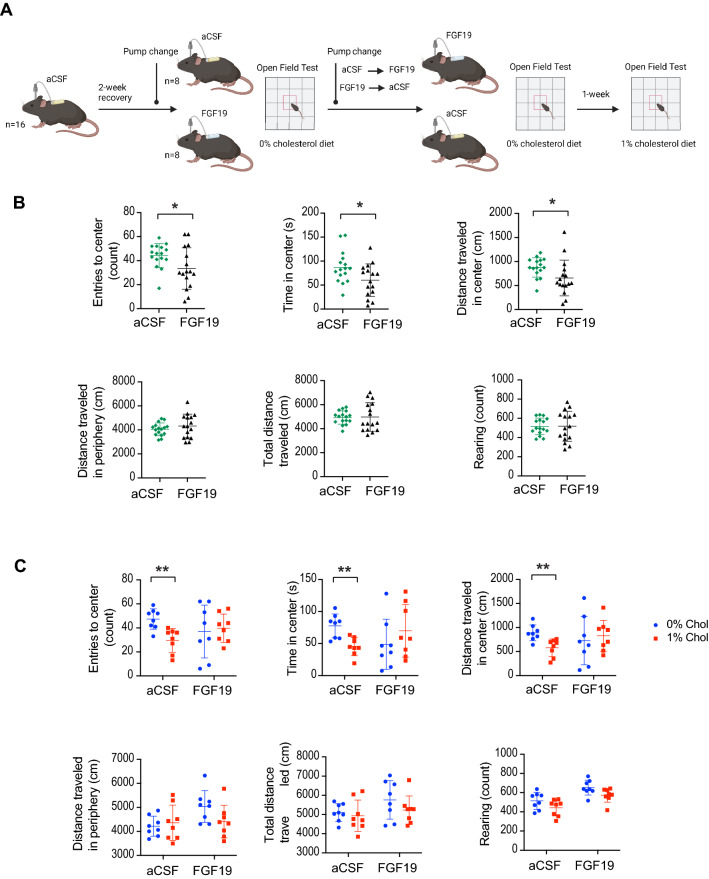
Dietary cholesterol and FGF19 in the mouse brain suppresses exploratory behavior. **A** 5-month-old male C56BL/6J mice (n = 16) underwent ICV osmotic minipump placement with aCSF (Alzet 0.5 µl /h) and were given 2-week recovery period. ICV pumps were then replaced with infusion of either FGF19 (15 ng/0.5 µl/h) or aCSF. Mice were placed into neurobehavioral open field testing chamber after being *ad lib* fed with a cholesterol-free diet (Teklad 2018) for 72 h. Using a cross-over design, mice underwent another ICV pump replacement and were given ICV infusion of either FGF19 or aCSF via osmotic minipump and placed *on ad lib* cholesterol-free diets 72 h prior to open field testing. One week later, mice were *ad lib* fed with the same diet supplemented with 1% cholesterol for 72 h and completed open field testing. **B** Open field testing results comparing mice receiving aCSF versus FGF19 infusion while on cholesterol-free diets. **C** Open field testing results comparing mice on cholesterol-deficient vs high cholesterol diet. *p ≤ 0.05. **p ≤ 0.01. ***p ≤ 0.001 by student *t*-test for (**B**) and 2-WAY ANOVA for (**C**)

We next evaluated if dietary cholesterol and FGF19 may affect exploratory behaviors in mice by using the open field test. To do so, wildtype C57BL6 mice, maintained on cholesterol-free chow (Teklad 2018), were cannulated in the lateral ventricle of the brain and infused with aCSF via subcutaneous osmotic minipumps (Fig. 4A). After 2 weeks of recovery, mice were divided into 2 weight-matched groups, and the minipumps were replaced with infusion of either aCSF or FGF19 (15 ng/0.5 µl/h). Mice then underwent open field testing. Next, in a cross-over experimental design, the osmotic minipumps were replaced with new pumps filled with either FGF19 or aCSF in a reciprocal fashion, and mice were then placed in open field testing (Fig. 4A). Mice that were infused with FGF19 intracerebroventricular (i.c.v.) showed significantly fewer entries to the center, spent less time, and traveled less distance in the center of the open field (Fig. 4B). FGF19 i.c.v. infusion did not affect the total distance traveled in the periphery, total distance, and rearing activity (Fig. 4B). These results suggest that central infusion of FGF19 may have an anxiogenic effect and reduce exploratory behaviors without altering total locomotor activity.

To further evaluate if dietary cholesterol may exert any effects on exploratory or anxiety-like behaviors, the aforementioned mice were given a 1-week to rest. The mice with functioning osmotic pumps were then provided with the same diet supplemented with 1% cholesterol for 72 h (Fig. 4A) and subsequently tested in the open field. The presence of dietary cholesterol led to reduced number of entries into the center, less time spent, and less distance traveled in the center of the open field in aCSF-infused control mice compared to the same aCSF-infused mice on cholesterol-free diet. Notably, the anxiolytic effects of cholesterol-free diet compared with cholesterol-rich diet were abolished by i.c.v. infusion of FGF19 (Fig. 4C). Distance in the periphery, total distance traveled, and rearing activity were not significantly different (Fig. 4C). Together, these experiments suggest that dietary cholesterol or central infusion of FGF19 suppresses exploratory behaviors, and that the anxiogenic effects of dietary cholesterol may be mediated by the effect of FGF19 in the mouse brain.

## Discussion

Feeding is a multi-step process whereby foraging for food precedes food consumption. Foraging is fundamental for survival in an environment where food is often scarce and unpredictable. Thus, these behaviors are well conserved and possessed by species across the animal kingdom including humans [33]. In this study, we present evidence that dietary cholesterol modulates locomotor activity and exploratory behavior, which are mediated in part by AgRP. We further show that dietary cholesterol stimulates intestinal production of FGF15/19, a postprandial hormone, which acts in the brain to exert anxiogenic effects and reduce exploratory behaviors.

Foraging behaviors and food consumption are tightly linked but can be uncoupled. Food deprivation is a potent driver for food-seeking behaviors as well as food consumption. In mice that are housed under standard laboratory condition, fasting markedly stimulates locomotor activity, which subsides upon refeeding. AgRP expression is markedly up-regulated by food deprivation. In Siberian hamsters, fasting or treatment with AgRP robustly increases foraging and food hoarding but has less effects on food intake [34–37]. Conversely, knockdown of AgRP in Siberian hamsters reduces food hoarding without affecting food intake [38]. These results suggest that AgRP triggers the search for food in this species, and once they find it, hoarding predominates over eating [37]. Our results show that AgRP is necessary for locomotor activity but not for food consumption in mice. Our study also shows that these behaviors can be influenced by the cholesterol content in the diet.

Foraging behaviors are influenced by a number of environmental factors. Our results indicate that fasting-induced increase in locomotor activity manifests only in the dark cycle but not in the light cycle, suggesting that hunger, when coupled with the anxiolytic effects of a dark environment, promotes foraging behaviors. On the other hand, our study shows that consumption of dietary cholesterol stimulates the expression of intestinal FGF15/19, which produces anxiogenic effects and suppresses exploratory activity. As production of FGF15/19 is stimulated by the postprandial release of bile acids, the effects of dietary cholesterol and FGF15/19 on exploratory activity indicate that a satiated state attenuates exploratory activity. On the other hand, the lack of dietary cholesterol or FGF15/19 may induce an anxiolytic state, which facilitates foraging activity. Consistent with this notion, activation of AgRP neurons exert anxiolytic effects [18]. Given that FGF19 suppresses *Agrp* and *Npy* expression and inhibits AgRP neuronal activity [27], these data suggest that FGF19 may exert anxiogenic effects by acting on multiple components of AgRP neurons. Thus, foraging behaviors are influenced by dietary, hormonal and environmental factors, and cholesterol-FGF15/19-AgRP regulatory axis is involved in this regulation.

**Fig. 5:**
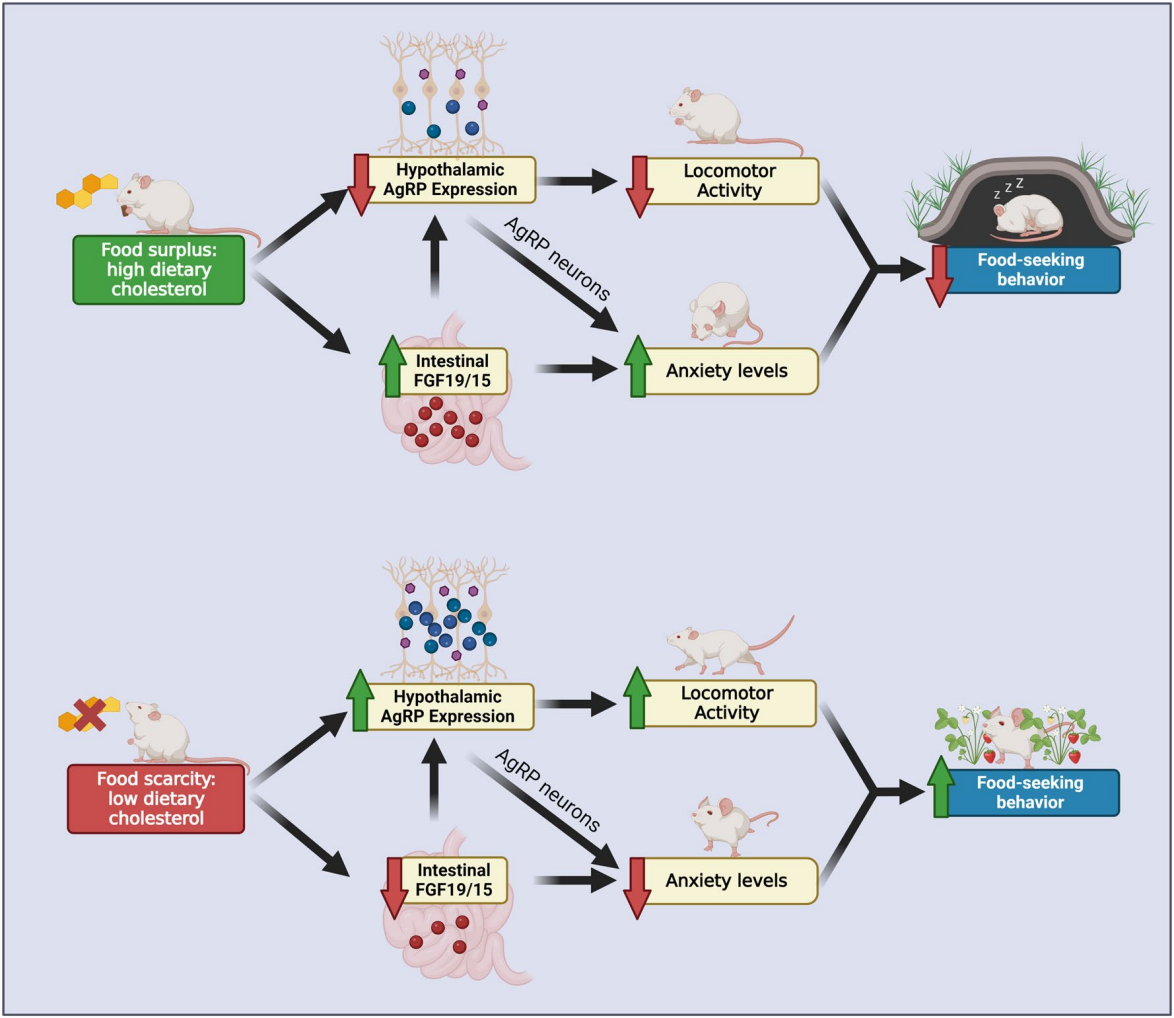
Graphical Abstract—Dietary cholesterol modulates locomotion and exploratory behavior through AgRP neuropeptide and intestinal fibroblast growth factor 15/19. In nature, dietary cholesterol comes from animal-based food. Thus, the presence of high dietary cholesterol may signal a state of energy surplus to the brain, in part via stimulation of intestinal FGF15/19 and suppression of hypothalamic AgRP expression, as well as alteration of other components of AgRP neurons. These effects lead to increased anxiogenic levels and decreased locomotor activities, thereby reducing food-seeking behaviors. On the contrary, when dietary cholesterol is depleted from the diet, it signals a state of food shortage, in part via suppression of intestinal FGF15/19 and stimulation of hypothalamic AgRP expression, as well as alteration of other components of AgRP neurons. These effects result in reduced anxiogenic levels and increased locomotor activities, thereby enhancing food-seeking behaviors. Green arrows signify upregulation and red arrows signify downregulation

In nature, dietary cholesterol comes from animal-based food. Thus, the presence of high dietary cholesterol may signal a state of energy surplus to the brain, in part via FGF19, which triggers adaptive down-regulation of AgRP neuropeptide expression, thereby reducing food seeking behaviors. On the contrary, when dietary cholesterol is depleted from the diet, it signals a state of food shortage, leading to enhanced locomotion and forage-like behaviors (Fig. 5). Understanding the genetic, dietary and hormonal modulators of foraging behaviors may help understand the complex feeding behaviors and shed light on the etiology of human obesity.

## Conclusions

Experiments in this study show that dietary cholesterol suppresses locomotor activity and foraging-like behaviors, and this regulation is in part mediated by AgRP. Dietary cholesterol or central infusion of FGF19 suppresses exploratory behaviors, and the anxiogenic effects of dietary cholesterol may be mediated by the effect of FGF19 in the mouse brain. This study suggests that dietary cholesterol and intestinal hormone FGF15/19 signal a satiating state to the brain, thereby suppressing foraging-like behaviors.

## Research design and methods

### Mice and diets

All animal care and experiments were approved by the University of California at San Francisco Institutional Animal Care and Use Committee. All experiments were performed using male mice, unless stated otherwise. Mice were housed in a barrier facility with 12 h light–dark cycles, and fed a mouse chow (LabDiet #5058), unless stated otherwise. C57BL/6J mice were purchased from the Jackson Laboratory, while *Agrp*^*–/–*^ mice were described previously [39]. Whenever possible, littermates were compared to account for variabilities in genetic background and litter dynamics. The macronutrient composition of different diets is described in each experiment. Cholesterol-free chow diet (Teklad 2018) and the matching diet with 1% supplemented cholesterol (TD. 02026) were manufactured by Envigo. To make custom cholesterol-free high-fat diet, TD8122 Basal Mix (Harlan Teklad, Madison, Wisconsin) was ground and mixed with pure virgin olive oil (CAS: 8001-25-0). Addition of cholesterol (Sigma-Aldrich St. Louis, MO, CAS: 57-88-5) to the diets was accomplished by dissolving it first in olive oil and then mixing it in with the ground diet.

### Body composition and metabolic measurements

Metabolic studies and body composition measurements were performed at the UCSF Mouse Metabolism Core, as previously described [40]. Briefly, body composition was measured using magnetic resonance imaging (EchoMRI-3in1 system, EchoMRI, Houston, TX, USA). Indirect calorimetry, locomotor activity, food intake, and energy expenditure were measured using a 16-chamber comprehensive lab animal monitoring system (CLAMS; Columbus Instruments, Inc.). During metabolic monitoring, mice were single-housed for 3 days prior to commencement of measurement and allowed 1 day to acclimatize in CLAMS chambers. O_2_ consumption, CO_2_ production, food intake, and locomotor activity were measured by CLAMS. Measurements over multiple days were recorded and data from the first day were excluded from analysis. For fasting-induced feeding measurements, mice were fasted at 11am for a duration of 24 h.

### Gene expression analysis

RNA isolation from mouse hypothalamic and intestinal tissues was performed using the RNeasy plus mini kit (Qiagen). qPCR was performed using Taqman gene expression assay probes: *Agrp*, Mm00475829_g1; *Npy*, Mm00445771_m1; *Pomc*, Mn00435874_m1, *Fgf15*, Mm00433278_m1 and *Gcg*, Mm01269055_m1.

### Intracerebroventricular infusion with osmotic pumps

In a single surgical procedure, 5-month-old mice were anesthetized with ketamine and xylazine (45 and 5 mg/kg, respectively) supplemented with isofluorane inhalation. Mice were mounted on a stereotaxic apparatus (model 1900; David Kopf Instruments) and implanted with an Alzet guide cannula (Durect) into the right lateral cerebroventricle (anteroposterior,—0.3 mm to bregma; lateral, + 1.0 mm to bregma; and dorsoventral,—2.5 mm below skull). The cannula was connected to an osmotic minipump (flow rate of 0.15 µl/h; Alzet model 2006; Durect) via a 50 mm-long vinyl tubing (inner diameter 1.22 mm, Durect). Each minipump was filled with artificial cerebrospinal fluid (aCSF). Pumps connected to the intracerebroventricular cannulae were primed overnight at 37 °C in 0.9% saline. The minipump was then implanted subcutaneously in the back posterior to scapulae. Mice were then housed singly and monitored for body weight, body composition, and food intake. All mice were allowed to recover for 2 weeks before experiments began. After the recovery period, each mouse underwent another surgical procedure where the osmotic minipump was replaced with a new minipump filled with either aCSF or FGF19 (15 ng/0.5 µl/h, Phoenix Pharmaceuticals, Inc), which were primed overnight at 37 °C in 0.9% saline. Mice then underwent open field testing. Two weeks later, the mice underwent a second osmotic minipump replacement such that those who initially received aCSF were switched to osmotic pumps filled with FGF19 and vice versa (cross-over study design). After each pump replacement, residual volumes in the pumps were measured to ensure that proper infusion took place.

### Open field testing

On the day of the open field testing (OFT), mice were transported to the behavioral testing room and allowed to habituate for at least 30 min before experiments. OFTs were performed in a brightly lit room. Mice were placed in the center of the open field (50 cm × 50 cm plexiglass chamber with 32 infrared photobeams), and their exploratory behaviors were tracked for 10 min and quantified using MotorMonitor software (Kinder Scientific, Chula Vista, CA). The arena was cleaned with 70 percent ethanol between trials. The time spent in, entries to and distance traveled in the center zone, distance in the periphery, total distance traveled and rearing were analyzed for each mouse.

### Statistical analyses

Specific statistical tests for different experiments are described in figure legends. Briefly, two-tailed Student’s *t*-test was used to compare two independent groups of mice. Under conditions where two genotypes and multiple treatments/conditions were compared, two-way ANOVA with multiple comparisons was performed. In cases where the same animals were analyzed over time, repeated-measures two-way ANOVA was used. Statistical analyses were performed using Prism software (GraphPad Software, Inc, La Jolla, CA, USA). Differences were regarded as statistically significant if p* < 0.05.

## Supplementary Information

**Additional file 1: Figure S1. **AgRP deficiency alone does not affect exploratory behavior. Eight-to-nine month-old male *Agrp*^*+/+*^ (WT, n=7) and *Agrp*^*–/–*^ (KO, n=9) mice were *ad lib* fed with a chow diet (Teklad 2018) for 72 hours and then placed into open field test. WT and KO mice were compared by student *t*-test.

## Data Availability

All data generated or analyzed during this study are included in this published article.

## References

[1] Couper R (1999). Prader-Willi syndrome. J Paediatr Child Health.

[2] Young J, Zarcone J, Holsen L, Anderson MC, Hall S, Richman D (2006). A measure of food seeking in individuals with Prader-Willi syndrome. J Intellect Disabil Res.

[3] Dimitropoulos A, Feurer ID, Roof E, Stone W, Butler MG, Sutcliffe J (2000). Appetitive behavior, compulsivity, and neurochemistry in Prader-Willi syndrome. Ment Retard Dev Disabil Res Rev.

[4] Doulla M, McIntyre AD, Hegele RA, Gallego PH (2014). A novel MC4R mutation associated with childhood-onset obesity: a case report. Paediatr Child Health.

[5] Turner L, Gregory A, Twells L, Gregory D, Stavropoulos DJ (2015). Deletion of the MC4R gene in a 9-year-old obese boy. Child Obes.

[6] Seligman HK, Bindman AB, Vittinghoff E, Kanaya AM, Kushel MB (2007). Food insecurity is associated with diabetes mellitus: results from the National Health Examination and Nutrition Examination Survey (NHANES) 1999–2002. J Gen Intern Med.

[7] Tester JM, Lang TC, Laraia BA (2016). Disordered eating behaviours and food insecurity: a qualitative study about children with obesity in low-income households. Obes Res Clin Pract.

[8] Poll KL, Holben DH, Valliant M, Joung HD (2018). Food insecurity is associated with disordered eating behaviors in NCAA division 1 male collegiate athletes. J Am Coll Health.

[9] Hossfeld LH, Rico Mendez G (2018). Looking for food: food access, food insecurity, and the food environment in rural Mississippi. Fam Community Health.

[10] Aponte Y, Atasoy D, Sternson SM (2011). AGRP neurons are sufficient to orchestrate feeding behavior rapidly and without training. Nat Neurosci.

[11] Krashes MJ, Koda S, Ye C, Rogan SC, Adams AC, Cusher DS (2011). Rapid, reversible activation of AgRP neurons drives feeding behavior in mice. J Clin Invest.

[12] Luquet S, Perez FA, Hnasko TS, Palmiter RD (2005). NPY/AgRP neurons are essential for feeding in adult mice but can be ablated in neonates. Science.

[13] Chen Y, Essner RA, Kosar S, Miller OH, Lin YC, Mesgarzadeh S (2019). Sustained NPY signaling enables AgRP neurons to drive feeding. Elife.

[14] Engstrom Ruud L, Pereira MMA, de Solis AJ, Fenselau H, Bruning JC (2020). NPY mediates the rapid feeding and glucose metabolism regulatory functions of AgRP neurons. Nat Commun.

[15] Dietrich MO, Zimmer MR, Bober J, Horvath TL (2015). Hypothalamic Agrp neurons drive stereotypic behaviors beyond feeding. Cell.

[16] Jikomes N, Ramesh RN, Mandelblat-Cerf Y, Andermann ML (2016). Preemptive stimulation of AgRP neurons in fed mice enables conditioned food seeking under threat. Curr Biol.

[17] Padilla SL, Qiu J, Soden ME, Sanz E, Nestor CC, Barker FD (2016). Agouti-related peptide neural circuits mediate adaptive behaviors in the starved state. Nat Neurosci.

[18] Li C, Hou Y, Zhang J, Sui G, Du X, Licinio J (2019). AGRP neurons modulate fasting-induced anxiolytic effects. Transl Psychiatry.

[19] Nelson NG, Wu L, Maier MT, Lam D, Cheang R, Alba D (2022). A gene-diet interaction controlling relative intake of dietary carbohydrates and fats. Mol Metab.

[20] Goldstein JL, Brown MS (1990). Regulation of the mevalonate pathway. Nature.

[21] Jurevics H, Hostettler J, Barrett C, Morell P, Toews AD (2000). Diurnal and dietary-induced changes in cholesterol synthesis correlate with levels of mRNA for HMG-CoA reductase. J Lipid Res.

[22] Chiang JY (2009). Bile acids: regulation of synthesis. J Lipid Res.

[23] Schaap FG (2012). Role of fibroblast growth factor 19 in the control of glucose homeostasis. Curr Opin Clin Nutr Metab Care.

[24] Morton GJ, Matsen ME, Bracy DP, Meek TH, Nguyen HT, Stefanovski D (2013). FGF19 action in the brain induces insulin-independent glucose lowering. J Clin Invest.

[25] Ryan KK, Kohli R, Gutierrez-Aguilar R, Gaitonde SG, Woods SC, Seeley RJ (2013). Fibroblast growth factor-19 action in the brain reduces food intake and body weight and improves glucose tolerance in male rats. Endocrinology.

[26] Fu L, John LM, Adams SH, Yu XX, Tomlinson E, Renz M (2004). Fibroblast growth factor 19 increases metabolic rate and reverses dietary and leptin-deficient diabetes. Endocrinology.

[27] Marcelin G, Jo YH, Li X, Schwartz GJ, Zhang Y, Dun NJ (2014). Central action of FGF19 reduces hypothalamic AGRP/NPY neuron activity and improves glucose metabolism. Mol Metab.

[28] Lan T, Morgan DA, Rahmouni K, Sonoda J, Fu X, Burgess SC (2017). FGF19, FGF21, and an FGFR1/beta-klotho-activating antibody act on the nervous system to regulate body weight and glycemia. Cell Metab.

[29] Wilson BD, Bagnol D, Kaelin CB, Ollmann MM, Gantz I, Watson SJ (1999). Physiological and anatomical circuitry between Agouti-related protein and leptin signaling. Endocrinology.

[30] Teske JA, Perez-Leighton CE, Billington CJ, Kotz CM (2014). Methodological considerations for measuring spontaneous physical activity in rodents. Am J Physiol Regul Integr Comp Physiol.

[31] Heinz DE, Schottle VA, Nemcova P, Binder FP, Ebert T, Domschke K (2021). Exploratory drive, fear, and anxiety are dissociable and independent components in foraging mice. Transl Psychiatry.

[32] Seibenhener ML, Wooten MC (2015). Use of the open field maze to measure locomotor and anxiety-like behavior in mice. J Vis Exp.

[33] Koster J, McElreath R, Hill K, Yu D, Shepard G, van Vliet N (2020). The life history of human foraging: cross-cultural and individual variation. Sci Adv.

[34] Wood AD, Bartness TJ (1996). Food deprivation-induced increases in hoarding by Siberian hamsters are not photoperiod-dependent. Physiol Behav.

[35] Bartness TJ, Clein MR (1994). Effects of food deprivation and restriction, and metabolic blockers on food hoarding in Siberian hamsters. Am J Physiol.

[36] Teubner BJ, Keen-Rhinehart E, Bartness TJ (2012). Third ventricular coinjection of subthreshold doses of NPY and AgRP stimulate food hoarding and intake and neural activation. Am J Physiol Regul Integr Comp Physiol.

[37] Day DE, Bartness TJ (2004). Agouti-related protein increases food hoarding more than food intake in Siberian hamsters. Am J Physiol Regul Integr Comp Physiol.

[38] Thomas MA, Tran V, Ryu V, Xue B, Bartness TJ (2018). AgRP knockdown blocks long-term appetitive, but not consummatory, feeding behaviors in Siberian hamsters. Physiol Behav.

[39] Maier MT, Vilhelmsson A, Louie SM, Vagena E, Nomura DK, Koliwad SK (2018). Regulation of hepatic lipid accumulation and distribution by agouti-related protein in male mice. Endocrinology.

[40] Yulyaningsih E, Rudenko IA, Valdearcos M, Dahlen E, Vagena E, Chan A (2017). acute lesioning and rapid repair of hypothalamic neurons outside the blood-brain barrier. Cell Rep.

