# Supplementary figures and images for: Modulation of foraging-like behaviors by cholesterol-FGF19 axis
Source: Cell Biosci. 2023 Feb 2;13:20. doi: 10.1186/s13578-023-00955-2 (PMC9893607; doi:10.1186/s13578-023-00955-2)

# Supplementary Figure 1

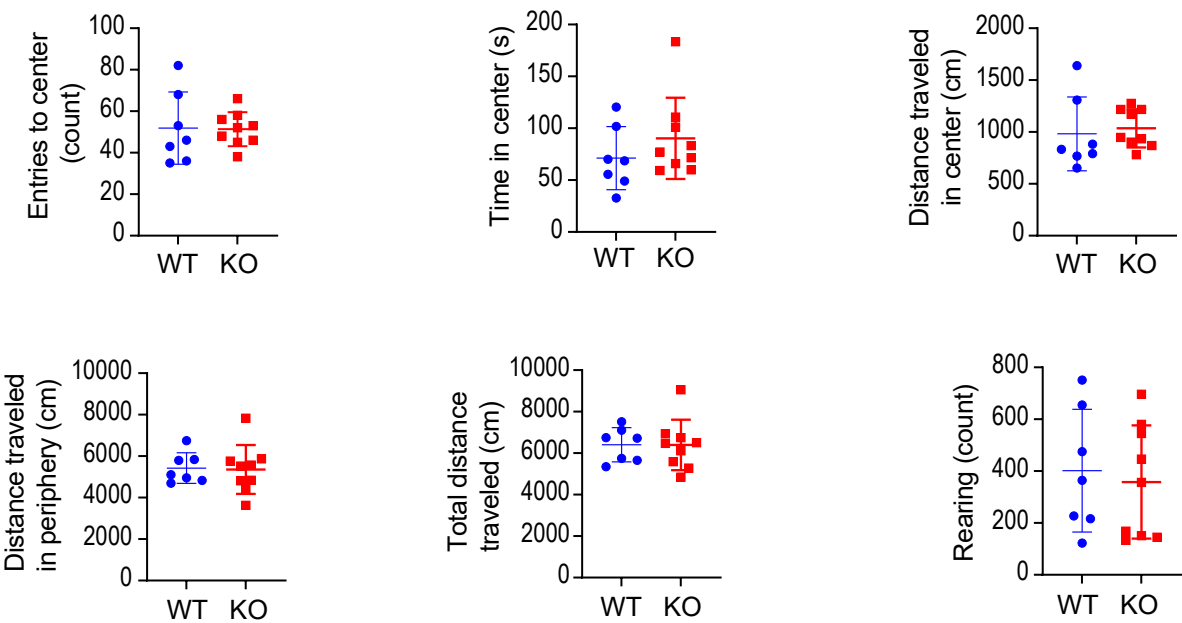

Supplement: Supplementary file 1 — Additional file 1: Figure S1. AgRP deficiency alone does not affect exploratory behavior. Eight-to-nine month-old male Agrp+/+ (WT, n=7) and Agrp–/– (KO, n=9) mice were ad lib fed with a chow diet (Teklad 2018) for 72 hours and then placed into open field test. WT and KO mice were compared by student t-test. [file 13578_2023_955_MOESM1_ESM.pdf]
